# The Same Thing from Different Points of View

**Published:** 1897-11

**Authors:** 


### THE SAME THING FROM DIFFERENT POINTS OF VIEW.

As it appeared in the August JOURNAL: "We veterinarians will bear in mind that Senate Bill No. 1063 still remains on the calendar of that body, awaiting a favorable opportunity to be called up for passage. This bill strikes right at the root of all original investigations of the Bureau of Animal Industry, and would likely be followed by similar efforts in many States, and thus would halt all progress in human and comparative science."

As reproduced in the (September 4th) *Spirit of the Times* without due credit being given: "Veterinarians should not forget that Senate Bill No. 1063 still remains on the calendar of that body. As soon as a favorable opportunity presents itself the bill will be called up for passage. This bill strikes right at the root of all original investigations in this subject, and would, in all probability, be followed by similar efforts in many States, which, if successful, would put an end to all progress in human and comparative medical science, and should be strenuously opposed."

As it appeared in the October number of the *American Veterinary Review*: "ANTI-VIVISECTION BILL.--Veterinarians should not forget that Senate Bill No. 1063 still remains on the calendar of that body. As soon as a favorable opportunity presents itself the bill will be called up for passage. This bill strikes right at the root of all original investigations in this subject, and would, in all probability, be followed by similar efforts in many States which if successful would put an end to all progress in human and comparative science and should be strenuously opposed."—*New York Spirit of the Times.*

The usually very alert eye of the junior editor of the *Review* could hardly have been turned on the recent issues of the JOURNAL as sharply as common.—ED.

---

THE experience of meat-inspector Schrieber, of Philadelphia, in finding hung up on a stall of a meat-dealer the diseased liver of a horse that had died some days before from causes that would have led to illness on the part of anyone eating the same, should be a lesson to all municipalities of the need of meat-inspection and the wisdom of establishing central abattoirs where all animals should be slaughtered that are intended for consumption as food.

